# Supplementary material for: DNA Sequencing of CD138 Cell Population Reveals TP53 and RAS-MAPK Mutations in Multiple Myeloma at Diagnosis
Source: Cancers (Basel). 2024 Jan 14;16(2):358. doi: 10.3390/cancers16020358 (PMC10813921; doi:10.3390/cancers16020358)
Supplement: Supplementary file 1 [file cancers-16-00358-s001.zip › cancers-2794591-supplementary.pdf]

cancers

Supplementary Table S1. Sequences of primers used for NGS

Primer	Sequence
**BRAF_exon_15_F**	TCGTCGGCAGCGTCAGATGTGTATAAGAGACAGCTGTTTTCCTTTACTTACTACACCTCA
**BRAF_exon_15_R**	GTCTCGTGGGCTCGGAGATGTGTATAAGAGACAGTAATCAGTGGAAAAATAGCCTCAAT
**NRAS_exon_1_F**	TCGTCGGCAGCGTCAGATGTGTATAAGAGACAGGCTCGCCAATTAACCCTGAT
**NRAS_exon_1_R**	GTCTCGTGGGCTCGGAGATGTGTATAAGAGACAGGGATCATATTCATCTACAAAGTGGTTCTG
**NRAS_exon_2_F**	TCGTCGGCAGCGTCAGATGTGTATAAGAGACAGGAAAACAAGTGGTTATAGATGGTGAAA
**NRAS_exon_2_R**	GTCTCGTGGGCTCGGAGATGTGTATAAGAGACAGTGATGGCAAATACACAGAGGAAG
**KRAS_exon_1_F**	TCGTCGGCAGCGTCAGATGTGTATAAGAGACAGTAAGGCCTGCTGAAAATGACTGAATA
**KRAS_exon_1_R**	GTCTCGTGGGCTCGGAGATGTGTATAAGAGACAGGCACCAGTAATATGCATATTAAAACAAGA
**KRAS_exon_2_F**	TCGTCGGCAGCGTCAGATGTGTATAAGAGACAGGAAACCTGTCTCTTGGATATTCTCG
**KRAS_exon_2_R**	GTCTCGTGGGCTCGGAGATGTGTATAAGAGACAGGCTTATTATATTCAATTTAAACCCACCTA
**HRAS_exon_1_F**	TCGTCGGCAGCGTCAGATGTGTATAAGAGACAGCCCCTGAGGAGCGATGACG
**HRAS_exon_1_R**	GTCTCGTGGGCTCGGAGATGTGTATAAGAGACAGCGCCAGGCTCACCTCTATA
**HRAS_exon_2_F**	TCGTCGGCAGCGTCAGATGTGTATAAGAGACAGGATGGGGAGACGTGCCTGTTGG
**HRAS_exon_2_R**	GTCTCGTGGGCTCGGAGATGTGTATAAGAGACAGTGTACTGGTGGATGTCCTCAAAAG

Supplementary Table S2. Demographic and clinical profile of study subjects.

Parameters	Total	Percent
**Female**	27	52,94 %
**Male**	24	47,05 %
**Age in years, median**	61 y	-
**IgG kappa**	14	27,45 %
**IgG lambda**	10	19,60 %
**IgA kappa**	4	7,84 %
**IgA lambda**	7	13,72 %
**IgD lambda**	1	1,96 %
**Free kappa**	9	17,64 %
**Free lambda**	4	7,84 %
**Cytogenetics (90,19 %)**		
t(14;16)	4	7,84 %
del17p13	18	35,29 %
del13q14	2	3,92 %
Gain 1q21	11	21,56 %
t(4;14)	9	17,64 %
t(11;14)	1	1,96 %
del11q	1	1,96 %
**Risk stratification**		
Standard-risk	21	41,17%
Intermediate-risk	8	15,68%
High-risk	22	43,13%
**ISS Classification**		
ISS-I	8	15,68 %

ISS-II	10	19,60 %
ISS-III	26	50,98 %
	**Induction therapy**	
VRd	4	7,84 %
KRd	4	7,84 %
DaraVTd	2	3,92 %
PAD	13	25,49 %
VelDex	1	1,96 %
CyBorD	15	29,41 %
Dara-CyBorD	13	11,76 %
No treatment	6	25,49 %
	**Maintenance therapy**	
Lenalidomide	10	19,60 %
KRd	3	5,88 %
LenDex	3	5,88 %
Bortezomib	2	3,92 %
Melphalan	2	3,92 %
VTd	1	1,96 %
**Refractory to the last treatment line**	**11**	**21,56 %**

Supplementary Table S3. Mutations and cytogenetic abnormalities identified in the refractory patient subgroup.

Refractory patient	Mutation	Cytogenetics	Refractory to induction therapy	Refractory to maintenance therapy
1	NRAS p.Q61K	t(4;14)		yes
2	BRAF p.V640G	del17p, gain 1q	yes	yes
3	KRAS p.G12A	del17p, t(4;14)	yes	
4	N/A	del17p		yes
5	BRAF p.D634N	gain 1q		yes
6	N/A	N/A	yes	yes
7	N/A	N/A	yes	
8	N/A	N/A		yes
9	N/A	N/A	yes	yes
10	N/A	N/A	yes	
11	N/A	N/A	yes	
